# Extracellular Y RNA fragments in gastrointestinal cancer and a proposed role for YBX1 in macrophage responses

**DOI:** 10.3389/fimmu.2026.1902611

**Published:** 2026-10-20

**Authors:** Xitan Wang, Fuli Wang, Wei Tian

**Affiliations:** Department of Oncology, Zibo Central Hospital, Zibo, Shandong, China

**Keywords:** extracellular vesicles, gastrointestinal cancer, m5C, macrophages, myeloid reprogramming, tumor microenvironment, Y RNA-derived fragments, YBX1

## Abstract

Tumor-derived extracellular vesicles (EVs) can alter macrophage responses, but the contribution of individual RNA cargoes remains difficult to establish. Y RNA-derived fragments are candidate mediators whose mechanisms after EV exposure have not been conclusively defined across physiological settings. This review examines reported EV-associated macrophage effects in gastrointestinal (GI) cancer, Y RNA studies from other diseases, and a possible role for RNA modifications and RNA-binding proteins. Separate experimental systems link YBX1 protein methylation to fragment sorting, damage-associated Y3 RNA methylation to YBX1 binding and PARP1 activity, and extracellular hY4 to monocyte responses through TLR7. These observations do not establish a continuous pathway in GI cancer. We propose that a sequence-defined Y RNA fragment could reach recipient macrophages, bind YBX1 in an m5C-sensitive manner, and alter a measurable molecular process before changing macrophage function. Whether this occurs, whether RNA methylation precedes or follows transfer, and whether the response supports tumor growth remain unresolved. A cardiac study using a synthetic Y RNA fragment reported reduced inflammatory-cell recruitment, illustrating that RNA responses can differ with disease and treatment context. The review distinguishes vesicle uptake from functional RNA delivery and considers endosomal sensing, other EV cargoes, and donor-cell effects as alternative explanations. Testing this hypothesis will require demonstrating functional delivery of a defined Y RNA fragment and YBX1-dependent macrophage effects in the same GI cancer model.

## 1 Introduction

Tumor-derived extracellular vesicles (EVs) are a possible source of signals that alter neighboring cells (1). Tumor-associated macrophages influence immune suppression and tumor progression (2), motivating investigation of the signals that change their function in gastrointestinal (GI) cancer. EV preparations contain proteins, lipids, and RNAs, and their effects on macrophages cannot be assigned to a particular RNA solely because that RNA is detected in the preparation or in exposed cells. This review asks which observations support RNA-dependent macrophage responses and what additional evidence would be required to implicate Y RNA-derived fragments.

MicroRNAs have been widely studied as candidate EV-associated signals in cancer (3), although efficient functional transfer cannot be assumed in every donor-recipient system (4). Other small non-coding RNAs include fragments of transfer RNAs, ribosomal RNAs, and Y RNAs that can arise independently of canonical microRNA biogenesis (5). Y RNA-derived fragments have been detected in extracellular RNA profiles (6, 7). Their Pol III origin and broader biological context are reviewed separately (8). Studies of Y RNA processing and extracellular release establish roles for RNA structure and associated proteins (9, 10). A separate DNA-damage study investigated Y3-fragment methylation and YBX1 binding (11). These observations do not establish a general modification-dependent mechanism for all extracellular Y RNA fragments.

The GI cancer literature reports macrophage responses to tumor EV preparations containing several candidate small RNAs, whereas Y RNA-specific myeloid evidence comes mainly from leukemia and non-tumor models. We examine these disease-specific observations before introducing a possible YBX1-dependent mechanism. Circulating Y RNA measurements are discussed separately because they do not establish a tumor source, delivery to macrophages, or a functional effect.

YBX1 warrants consideration because separate studies associate it with Y3-fragment activity after transfer, EV-associated RNA-binding proteins, and methylation-dependent sorting of an hY4 fragment (11–13). The proposed extension to GI macrophages requires direct testing at each step. The review therefore compares this hypothesis with endosomal RNA sensing and with effects of other EV components or soluble factors, rather than assuming that YBX1 explains all reported macrophage responses.

We distinguish three questions throughout: whether a Y RNA species is present in tumor-associated samples, whether it reaches a recipient compartment where it can act, and whether it causes a macrophage response through a defined mechanism. Evidence for one question does not answer the others. The tRNA-derived fragment tRF-3022b is included as a different small-RNA class with a cytokine-associated mechanism studied in a tumor-cell/macrophage system; it is not a Y RNA fragment.

## 2 Y RNA biogenesis modifications and RNA binding proteins

### 2.1 Y RNAs and the Ro60 ribonucleoprotein

Human Y RNAs are small, structured RNA polymerase III transcripts of approximately 80–110 nt that bind RO60, also known as SS-A/TROVE2 (14). Ro60 is a ring-shaped RNA-binding protein that recognizes structured or misfolded RNAs. Biochemical and bacterial studies support roles in RNA quality control and turnover, but the exact functions vary across organisms. Ro60-Y RNA complexes are also clinically relevant autoantigens in Sjogren syndrome and systemic lupus erythematosus (14).

### 2.2 Y RNA fragmentation differs from canonical microRNA biogenesis

Under cellular stress and apoptosis, Y RNAs can be processed into 5-prime and 3-prime fragments, with fragment sizes and dependencies varying by RNA species and cell system (9). The processing study identified structural requirements for RNY5 cleavage and a role for RO60, with RNase L contributing in some cells (9). A separate knockout analysis in HCT116 cells found that most tRNA-, Y RNA-, and rRNA-derived small-RNA signals were not reduced by loss of DROSHA, DGCR8, XPO5, or DICER (5). This supports a distinction from canonical microRNA biogenesis in that system, but does not establish that every Y RNA fragment in every tissue is independent of these factors. Because RO60 perturbation can change fragment production, EV-associated abundance must be interpreted alongside intracellular fragment abundance before a sorting effect is inferred.

### 2.3 Evidence for Y RNA methylation and YBX1 binding

Evidence for m5C-sensitive YBX1 binding comes from distinct experimental contexts. In the ionizing-radiation study by Shaw et al., synthetic Y3 5-prime ysRNA oligonucleotides showed damage-associated nuclear localization and NSUN2-dependent m5C modification in complementary mechanistic assays (11). The study connects Y RNA/YBX1 activity to PARP1-associated repair, but does not directly map the methylation history of a naturally transferred EV fragment. Separately, mRNA studies connect NSUN2/YBX1 activity with transcript stability (15, 16). The bladder-cancer study identifies W65 in the YBX1 cold-shock domain as important for m5C recognition (15). These mRNA findings do not define the activity of a short non-coding Y RNA fragment. The relevant methylation site, timing, reader requirement, and downstream response in GI macrophages remain unknown.

### 2.4 Regulation of EV loading

EV RNA profiles can differ from those of donor cells. Proteo-transcriptomic analysis identified co-enrichment of RNY3 and YBX1, while its IGF2BP2-related analysis concerned vault RNA; this does not establish IGF2BP2 as a Y RNA-binding or sorting factor (12). A separate lung cancer study demonstrated YBX1-methylation-dependent export of an hY4-derived fragment (13). In activated THP1-derived macrophages, Y RNA subtypes and RO60 showed altered intracellular distribution and extracellular release (10). Those measurements do not establish sequence-resolved fragment loading or RO60-dependent sorting. The responsible RNA species and regulatory mechanisms therefore require testing in each donor-cell system.

## 3 Evidence for EV associated macrophage responses

### 3.1 GI tumor EV studies and the limits of RNA transfer evidence

Studies in GI cancer report macrophage responses associated with EV preparations and candidate RNA cargoes. In CRC, miR-934 was linked to PTEN/PI3K-AKT signaling, macrophage M2-polarization readouts, and CXCL13-associated liver metastasis (17). In pancreatic cancer, hypoxic tumor EVs enriched in miR-301a-3p were linked to macrophage polarization through PTEN/PI3K-gamma (18). HCC studies reported effects of EV-associated miR-23a-3p on macrophage PD-L1 and CD8-positive T-cell activity (19), and of miR-21-5p on RhoB-associated polarization (20). These studies support investigation of EV-associated macrophage responses, but each requires assessment of RNA dose, carrier purity, delivery, and RNA-specific causality. None establishes a Y RNA-fragment/YBX1/m5C mechanism in GI cancer.

The mutant-p53 CRC study is retained with a specific qualification (21). The sequence commonly called miR-1246 overlaps a fragment of RNU2-1, the U2 small nuclear RNA, and can be detected by assays described as miR-1246 assays (22). Macrophages also contain endogenous U2 RNA. Increased signal in exposed macrophages, tissue co-localization, or responses to a synthetic mimic therefore cannot alone distinguish donor-derived transfer from endogenous RNA or establish canonical microRNA activity. Full fragment boundaries, orthogonal size-resolved assays, donor-specific tracing, and sequence-specific loss-and-rescue experiments are needed to assign transfer and function.

EV uptake and functional RNA delivery are separate measurements. Albanese et al. found that EV interaction with recipient cells did not generally produce efficient functional miRNA transfer in their experimental systems (4). This does not exclude all EV-RNA activity, but it prevents efficient transfer from being treated as a default assumption. For Y RNA studies, absolute cargo copies, exposure dose, and recipient-compartment access must be quantified. EV-enriched and EV-depleted fractions, appropriate soluble-factor controls, and RNase treatment with and without membrane disruption can help assess carrier association. Donor-specific labeling must control for free label, precursor carryover, and labeling-induced changes in RNA behavior.

### 3.2 A tRNA fragment associated with cytokine signaling in CRC

In CRC, tRF-3022b is a tRNA-derived fragment detected in tumor tissue and plasma exosomes (23). Its knockdown increased M2-polarization readouts in the experimental tumor-cell/macrophage system. The study investigated interactions involving LGALS1 and MIF, including RNA-protein binding assays using CRC-cell and THP1-cell material. These findings should not be reduced to an exclusively donor-cell mechanism. They also do not establish that native EVs deliver the fragment to macrophages or that recipient YBX1 recognizes it. The example therefore concerns a different small-RNA class and cytokine-associated regulation, with a direction and mechanism that must remain distinct from the proposed GI Y RNA pathway.

### 3.3 Y RNA studies in myeloid cells outside GI cancer

For Y RNA species, the closest myeloid evidence discussed here comes from systems outside the gastrointestinal tract. In chronic lymphocytic leukemia (CLL), Haderk et al. linked tumor EV-associated hY4 to cytokine release and PD-L1 expression through TLR7 (24). A later study reported preferential uptake of B-cell-receptor-stimulated CLL EVs by monocytes and hY4-associated CCL24 expression and nurse-like differentiation (25). These studies provide a reason to investigate Y RNA responses in myeloid cells, but neither establishes m5C-sensitive recognition by recipient-cell YBX1. Reported hY4 species must also be distinguished from sequence-defined shorter fragments before comparing mechanisms across studies.

YF1 is a 56-nt Y RNA-derived fragment originally identified in cardiosphere-derived EVs. In a transgenic mouse model of hypertrophic cardiomyopathy, intravenous administration of synthetic YF1 reduced inflammatory-monocyte mobilization and macrophage accumulation and altered macrophage transcriptional responses (26). Because the intervention used the synthetic RNA on its own, this study does not establish physiological delivery by native EVs. A separate nematode study reported transfer of EV-associated small RNAs to mammalian cells and suppression of type-2 innate responses (27); it did not isolate a Y RNA-specific contribution to the immune phenotype.

These non-GI observations provide a rationale for testing Y RNA-dependent macrophage responses in GI tumors, but they do not predict the receptor or the direction of the response. hY4-related RNAs appear in both the lung cancer sorting study and leukemia myeloid studies (13, 24, 25), yet their fragment boundaries, modification states, and biological contexts cannot be treated as interchangeable. An immunosuppressive response is one candidate outcome suggested by the GI EV literature, not an established or inevitable property of Y RNA cargo. The TLR7 mechanism provides a demonstrated sensing alternative, while the synthetic-YF1 study illustrates a different RNA intervention and disease context. **Figure 1** and **2** summarize these distinctions. In **Figure 1** and **2**, P1 denotes the untested GI tumor-to-myeloid Y RNA/YBX1 hypothesis specified in Section 6. The separate lung-cancer, cultured-cell DNA-repair, leukemia, and cardiac examples provide context; they do not demonstrate a single continuous pathway in GI cancer.

**Figure 1:**
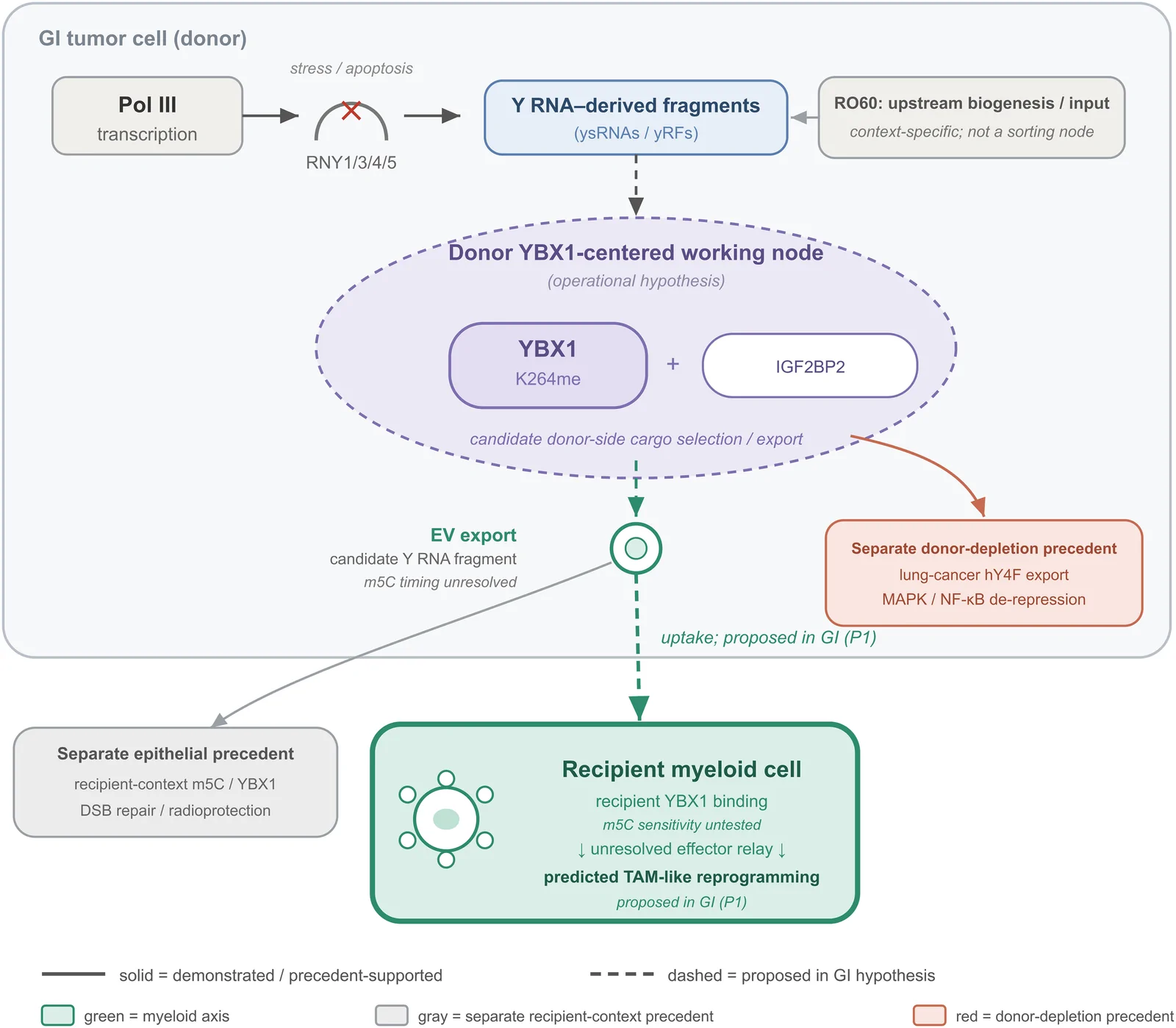
Proposed Y RNA-fragment export and myeloid response in GI cancer. P1 denotes the untested GI hypothesis defined in Section 6. The enclosing GI donor-cell box identifies the proposed setting; it does not locate all supporting experiments in GI cancer. Solid arrows summarize relationships supported in their original experimental contexts, whereas dashed arrows indicate proposed or unresolved connections. Box borders indicate visual grouping, not evidence strength. RO60 denotes an upstream influence on fragment accumulation, not an established sorting factor (9). K264me denotes YBX1 protein methylation, supported for hY4-fragment export in a lung-cancer model, and is distinct from RNA m5C (13). IGF2BP2 is shown only as an EV-associated candidate; the cited study does not demonstrate Y RNA binding or sorting by this protein (12). Its placement and the plus sign do not establish co-binding or a YBX1-containing complex. The green uptake route requires demonstration of donor-derived RNA access to the relevant intracellular compartment; uptake alone does not establish functional delivery. Recipient YBX1 binding, m5C dependence, the effector step, and the TAM-like outcome remain predictions. The orange branch depicts the separate lung-cancer donor-depletion example (13). The gray epithelial comparator refers to cultured-cell ionizing-radiation experiments and complementary synthetic-Y3 assays involving NSUN2, YBX1, and PARP1 (11); it does not show GI macrophage reprogramming or trace the methylation history of native EV cargo. RNA m5C timing before or after export remains unresolved. EV, extracellular vesicle; GI, gastrointestinal; TAM, tumor-associated macrophage.

**Figure 2:**
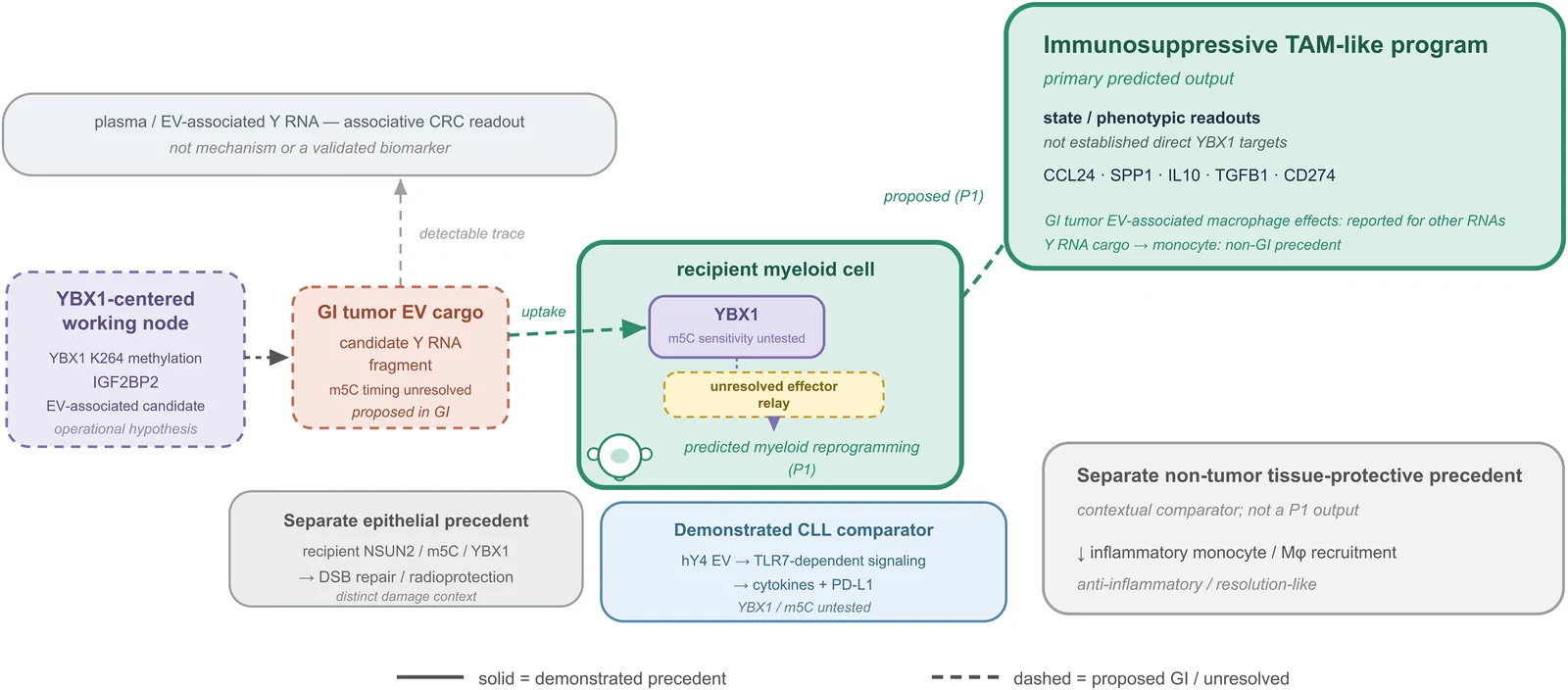
Proposed myeloid outcomes and separate experimental comparators. P1 is the untested GI Y RNA/YBX1 hypothesis defined in Section 6. Dashed connections require validation; solid relationships refer only to the original experimental context. Box borders indicate visual grouping, not evidence strength. The green TAM-like program is a candidate outcome, not an established effect of GI Y RNA cargo. CCL24, SPP1, IL10, TGFB1, and CD274 are phenotypic readouts, not established direct YBX1 targets; immune suppression requires functional testing. The revised label “GI tumor EV-associated macrophage effects: reported for other RNAs” refers to macrophage effects reported in selected GI EV/RNA studies (17–21). It does not establish efficient physiological RNA delivery, equivalent evidence across RNA classes, or a Y RNA mechanism. The tRF-3022b example discussed in Section 3.2 concerns a tRNA fragment and is not a Y RNA component of P1 (23). The blue CLL comparator represents hY4-associated TLR7-dependent cytokine and PD-L1 responses; neither recipient YBX1 nor m5C dependence was established (24). The gray DNA-repair comparator concerns a distinct irradiation setting (11). The separate tissue-protective comparator refers to synthetic 56-nt YF1 administration in cardiac disease, not native EV delivery or a demonstrated GI antitumor effect (26). The plasma/EV box indicates an association requiring carrier resolution, source attribution, and validation (38, 39); its dashed connection does not establish GI tumor origin. IGF2BP2 is shown only as an EV-associated candidate; the cited study does not demonstrate Y RNA binding or sorting by this protein (12). Uptake does not demonstrate functional delivery. CLL, chronic lymphocytic leukemia; CRC, colorectal cancer.

### 3.4 Macrophage heterogeneity and tumor immune context

Single-cell analysis of human CRC and corresponding mouse models identified macrophage subsets with different inflammatory and pro-angiogenic or tumor-associated transcriptional programs (28). A pan-cancer myeloid atlas likewise identified heterogeneous TAM populations with variable marker programs across cancer types (29). We therefore propose describing recipient responses using context-specific transcriptional and functional measurements rather than a binary M1/M2 classification. CCL24, SPP1, and PD-L1 are candidate readouts in the proposed experiments; no study reviewed here establishes that an EV-delivered Y RNA fragment produces a particular GI TAM subset through YBX1.

Any macrophage response should be interpreted alongside the baseline tumor immune environment (30). A change in CD163, CD206, or SPP1 expression alone does not demonstrate suppression of antitumor immunity. Studies should also measure macrophage effects on T-cell activation, proliferation, or cytotoxicity and account for pre-existing inflammatory and differentiation states. These functional data are needed before assigning therapeutic significance to an EV-associated change in macrophage markers.

### 3.5 DNA damage studies and their relevance to GI cancer

The key EV-transfer experiments in Shaw et al. used ionizing radiation, not ultraviolet irradiation (11). EVs from irradiated donors were applied to recipient cells, including recipients subsequently irradiated to assess radioprotection. Complementary assays linked Y3 5-prime ysRNA, NSUN2, YBX1, and PARP1 residency at DNA breaks. The reported endpoint concerns damage repair; it does not establish a change in macrophage differentiation or immune function. Moreover, several mechanistic assays used synthetic oligonucleotides, so they should not be described as direct tracking of methylation on naturally delivered EV cargo.

Acute irradiation in this model cannot be assumed to reproduce the duration, lesions, or cellular responses of GI tumor stress. For example, in human colorectal organoid models, 5-fluorouracil-induced pyrimidine imbalance led to DNA damage and cell death in actively proliferating p53-deficient tumor cells (31). This provides a GI-relevant stress context, but it does not show Y RNA export or macrophage signaling. No study reviewed here directly links GI tumor damage to YBX1-dependent enrichment of a defined Y RNA fragment in EVs, its functional delivery to macrophages, and subsequent macrophage reprogramming. Shared damage signaling supplies a reason to test this connection, not evidence that it occurs.

## 4 A proposed YBX1 dependent pathway in GI macrophages

### 4.1 Steps that require separate experimental tests

The hypothesis separates fragment biogenesis, donor-cell sorting, functional delivery, recipient-cell recognition, and downstream activity. Donor-cell YBX1 K264 methylation regulates hY4-fragment sorting in a lung cancer model (13); this protein modification must be distinguished from m5C modification of the RNA. IGF2BP2 is an EV-associated RBP candidate, but the cited proteo-transcriptomic study does not demonstrate Y RNA binding or sorting by this protein (12). RO60 loss affects fragment accumulation in the cited processing system (9), so changes in EV RNA after RO60 perturbation require comparison with intracellular substrate abundance. None of these donor-side findings establishes that GI tumor EVs contain the relevant methylated fragment or that recipient macrophages use YBX1 to respond. **Figure 1** summarizes the reported precedents and the proposed steps.

### 4.2 Functional delivery and recipient access

Shaw et al. observed enrichment of 5-prime Y1/Y3-derived fragments in EVs from irradiated HEK293T donors and reduced enrichment after donor-cell YBX1 knockout, consistent with a role in fragment packaging (11). Their EV-pretreatment experiments and complementary Y3 oligonucleotide experiments support a damage-associated RNA/YBX1/PARP1 mechanism in cultured-cell models. They do not establish the methylation state of the EV-associated fragment, direct modification of the same EV-derived molecule after transfer, or macrophage activity. Donor packaging, recipient access, RNA modification, and downstream effects therefore require separate measurements in a GI tumor-to-macrophage system.

Macrophages can internalize EVs through several uptake routes (32, 33), making them plausible recipients. These observations do not establish organ-specific targeting or cytosolic access for a particular RNA. Internalized material may remain in endosomes or be degraded. The proposed intracellular YBX1 mechanism requires delivery to the compartment containing the relevant YBX1 pool, whereas TLR7-dependent signaling can occur after endosomal access. No study discussed here establishes that a sequence-defined Y RNA fragment from a GI tumor reaches macrophages at a dose sufficient for m5C-sensitive YBX1 activity.

### 4.3 Candidate molecular effects in recipient macrophages

A key mechanistic question is how binding of a Y RNA fragment to recipient-cell YBX1 is translated into a myeloid transcriptional response. Rather than assuming a direct link to CCL24, SPP1, IL10, TGFB1, or CD274/PD-L1 expression, the proposed pathway can be viewed as a sequence of separable events: delivery of a sequence-defined fragment, m5C-sensitive binding to recipient-cell YBX1, a YBX1-dependent proximal molecular response, and subsequent myeloid-state reprogramming. The biochemical plausibility of m5C-sensitive YBX1 recognition is supported by studies of Y3-derived RNA and m5C-modified mRNAs (11, 15, 16), but the downstream effector step in myeloid cells remains unresolved. The listed genes are therefore treated as phenotypic readouts rather than presumed direct YBX1 targets.

One plausible mechanism is that the incoming fragment functions as an RNP scaffold or competitive RNA ligand that redistributes YBX1 among endogenous RNA and protein partners. Such redistribution could alter the stability or translation of YBX1-associated immune-regulatory transcripts and thereby reshape downstream myeloid programs. This possibility is proposed as a testable mechanism rather than an established function of Y RNA fragments. It can be examined through fragment-centered pull-down/proteomics, YBX1 eCLIP or RIP-seq, RNA half-life profiling, ribosome profiling or proteomics, and phospho-signaling analyses, ideally combined with YBX1 loss-and-rescue experiments and m5C-site controls.

These approaches would help distinguish a YBX1-dependent effector route from alternative RNA-sensing mechanisms. In particular, if fragment delivery induces a myeloid phenotype without a detectable YBX1-dependent proximal molecular response, endosomal RNA-sensing pathways should be considered separately. TLR7 is especially relevant because a Y RNA-to-myeloid signaling route has already been demonstrated through this pathway (24), while TLR8 may serve as a related RNA-sensing control.

### 4.4 Donor depletion and recipient responses

EV export can also affect the donor cell by reducing its intracellular pool of specific Y RNA fragments. When methylated YBX1 sorts a tumor-suppressive hY4-derived fragment into EVs, intracellular depletion of that fragment relieves its restraining effect on MAPK/NF-kappaB signaling (13). This donor-cell effect is distinct from the cultured-cell DNA-damage findings, which combine EV-transfer and synthetic-oligonucleotide experiments involving NSUN2/YBX1/PARP1 (11). Donor-cell depletion and recipient-cell responses should therefore be evaluated separately when assessing Y RNA-fragment export.

### 4.5 Alternative readers and sensing mechanisms

Several alternatives should be evaluated alongside YBX1. ALYREF is an m5C reader in mRNA export (34), SUMOylated hnRNPA2B1 can participate in the sorting of selected miRNAs (35), and m6A-dependent regulation is established for other RNA substrates (36). These findings motivate candidate tests but do not show that these proteins or modifications act on the GI Y RNA fragment proposed here. Extracellular Y RNAs can also engage endosomal RNA sensors (37). In CLL-associated experiments, hY4 induces cytokine and PD-L1 responses through TLR7 (24); this establishes a sensing mechanism without demonstrating or excluding an additional YBX1 contribution. TLR7 perturbation, with TLR8 included as a species- and cell-context-appropriate comparator, can distinguish endosomal sensing from intracellular reader activity. EV proteins or lipids, co-isolated soluble mediators, and donor-cell changes in cytokine secretion must also be tested as possible causes of macrophage responses.

## 5 Circulating Y RNA associations and their limitations

Circulating Y RNA changes have been reported in GI cancer, including elevated plasma Y RNAs in CRC (38) and Y RNA detection in paired plasma/EV profiling, with greater relative enrichment in whole-plasma cfRNA than in EV cfRNA (39). These observations belong to a wider biomarker literature with limitations in cohort size, selection, and clinical validation (40). Shared genetic loci for blood traits and cancer risk were enriched near Y RNA-related sequences; a separate plasma analysis found higher Y RNA levels before breast cancer diagnosis (41). These findings do not establish a GI-specific causal mechanism. Y RNA differences have been reported in glioblastoma plasma EVs and breast-cancer plasma (42, 43), whereas bladder-tumor tissue sequencing detected Y RNA-derived fragments with specific cleavage patterns (44). These findings show detectability and disease association, but the studies reviewed here do not establish that a circulating GI cancer Y RNA signal originates from tumor cells, acts on macrophages, or reflects a specific myeloid mechanism.

Circulating RNA measurements do not by themselves identify a functional EV cargo. Chevillet et al. reported low average copy numbers of individual microRNAs in exosomes (45); these measurements cannot be assumed to apply quantitatively to Y RNA fragments, which need their own absolute copy-number measurements. Albanese et al. found little functional miRNA delivery in the systems they tested despite EV-cell interactions (4). Reassessment of EV preparations also distinguished vesicles from non-vesicular extracellular material containing RNA-binding proteins (46). Carrier identity therefore requires experimental validation. The exRNAQC study directly measured miRNA and mRNA profiles and showed substantial effects of blood collection and purification procedures (47). It did not quantify the same effects for every Y RNA fragment, which requires its own validation. Blood-cell contributions also complicate interpretation of plasma and EV cfRNA profiles (39). A plasma association does not establish tumor origin, macrophage exposure, or a causal immune effect.

## 6 Experimental predictions and criteria for rejection

The principal prediction (P1 in **Figure 1** and **2**) specifies a series of experimentally separable events linking EV-mediated Y RNA-fragment delivery to myeloid-cell reprogramming. GI tumor-derived EVs would first need to deliver a sequence-defined Y RNA fragment to monocytes or macrophages, followed by m5C-sensitive recognition of the incoming fragment by recipient-cell YBX1. This interaction would then be expected to produce a measurable YBX1-dependent proximal molecular response before the emergence of an immunosuppressive TAM-like phenotype characterized by features such as increased CCL24, SPP1, IL10, TGFB1, or CD274/PD-L1 expression (17–21, 23–25, 28). These genes are best considered phenotypic readouts rather than presumed direct YBX1 targets. Experimental interruption at individual stages of this sequence would help define the contribution of each component; in particular, persistence of the myeloid program after recipient-cell YBX1 disruption would argue against a central requirement for YBX1, whereas persistence after perturbation of fragment m5C would indicate that the response is not dependent on the proposed m5C-reading mechanism.

Acute recipient-cell YBX1 depletion and rescue can test whether YBX1 is required, provided cell viability, differentiation, EV uptake, and baseline inflammatory responses are measured. Wild-type YBX1 can be compared with W65A, a mutant used in the bladder-cancer mRNA m5C-binding study (15), but W65A should not be assumed to affect only m5C recognition; expression, localization, and general RNA binding require controls. Chemically matched modified and unmodified fragments should be compared, with sequence mutants evaluated separately because they can alter RNA structure as well as methylation. Direct fragment-YBX1 binding and an early molecular response must precede interpretation of later macrophage markers. Parallel perturbation of TLR7-MYD88 signaling and appropriate TLR8 controls would test an alternative sensing mechanism (24).

Delivery experiments should quantify RNA per particle and per recipient cell and compare doses with measured tumor or plasma exposure. A donor-specific tracing strategy should be validated against endogenous recipient RNA, free labels, and non-vesicular carriers. Uptake measurements should be paired with compartment-specific localization or a validated functional-delivery assay. RNA-selective donor depletion and add-back at the measured dose should be combined with EV-depleted conditioned medium, soluble-factor controls, and assessments of other cargo changes. If an effect occurs only after direct transfection or very high EV doses, it does not establish physiological EV delivery. If matched EV-depleted fractions reproduce the response, vesicle-specific attribution is weakened.

Donor-side sorting can be assessed by perturbing YBX1 K264 methylation or candidate RBPs and quantifying both intracellular fragments and EV-associated fragments. EV output, particle-associated RNA abundance, donor viability, and cell-death-associated release should be measured separately. A change in EV RNA after a perturbation that also changes intracellular RNA or EV number does not by itself establish selective sorting. If the proposed determinants can be altered without changing fragment enrichment or recipient responses under matched conditions, their proposed sorting role would be weakened.

The effect of donor stress should be tested by comparing untreated GI tumor cells with defined damage or inflammatory conditions at matched viability. Each experiment should distinguish changes in fragment production, EV number, and copies per EV, and should quantify the dose delivered to recipient cells. Shared activation of a DNA-damage response is insufficient to infer the same export mechanism. Inflammatory cues can be examined as additional variables (48), without assuming that they regulate YBX1 methylation or Y RNA m5C in GI cancer.

A separate observational test is whether fragments identified in GI tumor EV preparations are detectable in patient plasma EV fractions and associated with myeloid infiltration or immune-suppressive features. Analyses should account for tumor burden, blood-cell composition, treatment, and sample handling. Even a correlation that persists after these adjustments would remain associative and would not establish tumor origin, delivery to macrophages, or reader-dependent causality.

PARP1-associated repair in the cultured-cell DNA-damage model is a separate outcome from macrophage reprogramming. A YBX1-dependent macrophage effect would require identification of its own downstream mechanism; PARP1 should be tested as a candidate only if recipient-cell measurements support its involvement.

PDAC and HCC are useful settings because suppressive myeloid populations are prominent and EV-associated macrophage effects have been reported (18–20, 49–51). CRC adds tumor EV studies, a distinct tRNA-fragment mechanism, and well-characterized TAM and MDSC biology (17, 21, 23, 52, 53). Gastric cancer offers another macrophage-rich setting, although the studies reviewed here provide less direct EV-RNA evidence (54). Selection of a model should also require detectable candidate fragments and a measurable macrophage response; an immunosuppressive microenvironment alone does not validate the hypothesis. **Table 1** summarizes disease contexts, and **Table 2** specifies the measurements needed before assigning RNA-specific causality.

**Table 1:** GI cancer models and macrophage readouts for testing the proposed pathway.

GI cancer	Myeloid context	Reported RNA findings	Functional readouts	Use and limitations
PDAC	Myeloid-rich, suppressive TME with generally limited responsiveness to checkpoint blockade (49).	Hypoxic PDAC EV preparations enriched in miR-301a-3p were associated with macrophage polarization through PTEN/PI3K-gamma (18).	M2 markers (CD163/CD206), Arg1, IL-10/TGF-β, and myeloid suppression linked to PDAC immune resistance.	Candidate setting with prominent myeloid suppression and reported EV-associated macrophage effects; Y RNA causality remains untested.
HCC	Inflammation-driven, TAM-abundant TME (50, 51)	HCC studies link EV-associated miR-23a-3p to macrophage PD-L1 and CD8-positive T-cell effects (19), and miR-21-5p to RhoB-associated polarization (20).	Macrophage PD-L1, IL-10/TGF-β, M2 markers, and CD8-positive T-cell suppression.	Candidate setting for functional immune readouts; neither a Y RNA mechanism nor general delivery efficiency is established.
CRC	TAM-related immune suppression and PMN-MDSC biology, including the CCL2-MDSC axis (52, 53).	Mutant-p53 EV study reports miR-1246-associated effects, with U2-fragment identity and endogenous recipient RNA requiring controls (21, 22); miR-934 study reports polarization and CXCL13 feedback (17); tRF-3022b was investigated in LGALS1/MIF-associated tumor-cell/macrophage experiments (23).	CD163/CD206, IL-10/TGF-β, CXCL13, CCL24, and CCL2-MDSC features.	Includes distinct small-RNA studies and a circulating Y RNA association (38); these do not demonstrate Y RNA delivery or YBX1-dependent macrophage activity.
Gastric cancer	TAM-associated progression and immune suppression involving cytokines such as IL-10 and TGF-beta (54).	No setting-specific EV-RNA-to-myeloid precedent was identified in the current evidence set.	M2 markers, IL-10/TGF-β, and TAM-associated progression signals.	Additional setting: relevant TAM biology but limited EV-RNA-to-myeloid evidence.

CRC, colorectal cancer; EV, extracellular vesicle; GI, gastrointestinal; HCC, hepatocellular carcinoma; MDSC, myeloid-derived suppressor cell; PDAC, pancreatic ductal adenocarcinoma; PMN-MDSC, polymorphonuclear myeloid-derived suppressor cell; TAM, tumor-associated macrophage; TME, tumor microenvironment.

**Table 2:** Experimental requirements for testing EV-associated Y RNA effects on macrophages.

Stage	Objective	Representative approaches and readouts
1 Fragment identity and carrier validation	Identify the sequence, abundance, carrier, and modification state of candidate Y RNA fragments	Resolve fragment ends and sizes; quantify absolute copies in donor cells and EV preparations; compare EV-enriched and non-vesicular fractions; apply RNase with and without membrane disruption and matched disruption controls; measure RNA m5C with conversion and structure controls plus an orthogonal method. Distinguish RNA methylation from YBX1 protein methylation.
2 Delivery and molecular recognition	Establish donor origin, recipient access, and an early molecular effect at a relevant dose	Use donor-specific tracing with free-label and precursor-carryover controls; quantify cargo per particle and recipient cell; validate compartmental access; compare EV-depleted medium and soluble fractions. Measure fragment-YBX1 binding, RNA or protein changes, and TLR7-MYD88 responses. Analyze donor sorting separately from recipient recognition.
3 Causal perturbation and alternatives	Distinguish a fragment-specific YBX1 pathway from other EV or soluble signals	Combine selective fragment depletion and dose-matched add-back with recipient YBX1 depletion and rescue; validate W65A expression, localization, and general RNA-binding controls. Compare chemically matched modified and unmodified RNA; test TLR7 and appropriate TLR8 controls. Measure donor viability, intracellular RNA, EV output, and cargo enrichment during sorting perturbations. Compare untreated and defined GI-relevant stress conditions.
4 Tissue and immune function	Determine whether the mechanism changes macrophage function in a GI tumor context	Pair single-cell and spatial macrophage profiling with *in vivo* biodistribution and measured exposure levels. Test effects on T-cell activation, proliferation, or cytotoxicity rather than relying on M2 markers alone. Treat plasma correlations as association and test whether findings persist across models and patient-derived systems.

## 7 Inflammation and the Cancer Evo-Dev framework

Chronic, non-resolving inflammation can promote cancer evolution through inflammatory remodeling, epigenetic reprogramming, and selection under immune pressure, as described in the Cancer Evo-Dev framework (48). Inflammation-associated cancers also contain suppressive myeloid populations, including tumor-associated macrophages and myeloid-derived suppressor cells (55, 56). These features are relevant to the TAM- and MDSC-rich microenvironments of PDAC, HCC, and CRC summarized in **Table 1**.

The Cancer Evo-Dev framework links chronic inflammation with mutation, selection, adaptation, and remodeling of the tumor environment (48). It does not identify the signals that maintain particular macrophage states. Whether inflammatory conditions alter YBX1 modification, fragment export, or recipient RNA recognition must be measured directly in GI models. The donor-cell benefit of hY4-fragment export in lung cancer (13) also remains distinct from any proposed effect on macrophages.

Reduced inflammatory-cell recruitment can have different consequences in cancer and cardiac disease. The cardiac study reported a tissue-protective response after administration of synthetic YF1 (26), but it does not demonstrate an antitumor program or a treatment for GI cancer. Likewise, the tRF-3022b study concerns a different RNA class and LGALS1/MIF-associated observations in a tumor-cell/macrophage system (23). Dose, exposure duration, RNA species, and recipient differentiation should therefore be treated as experimental variables, not as explanations assumed after an outcome is observed.

## 8 Limitations

The proposed YBX1 pathway has not been demonstrated in GI tumor-associated macrophages. Its supporting observations come from different RNA species, cell types, and experimental conditions. GI tumor EV studies do not establish a Y RNA mechanism, leukemia hY4 studies establish a TLR7 route without resolving a YBX1 requirement, and the cultured-cell DNA-damage study does not establish macrophage reprogramming (11, 17–21, 23–25). Evidence for an RNA in an EV preparation, uptake of a vesicle label, or induction of a macrophage marker is insufficient to connect the complete pathway.

Major unresolved steps include the identity and abundance of the active fragment, its vesicular versus non-vesicular carrier, access to the appropriate recipient compartment, the timing and site of m5C modification, and a fragment-specific YBX1-dependent molecular effect. Endogenous recipient Y RNAs make donor attribution difficult. No study reviewed here directly links GI tumor damage to YBX1-dependent enrichment of a defined Y RNA fragment in EVs, its functional delivery to macrophages, and subsequent macrophage reprogramming. The absence of this evidence limits the present proposal to a mechanistic hypothesis.

Alternative explanations include TLR7 or other RNA sensors, proteins and lipids associated with EVs, soluble factors co-isolated with vesicles, and changes in donor-cell cytokine production. Other readers may also contribute, but their activity on the proposed fragment remains unproven. A negative YBX1 result would reject the YBX1-dependent explanation under the tested conditions without excluding all Y RNA or EV effects. These alternatives need direct comparisons in the same experimental system.

## 9 Conclusion

Current studies justify asking whether Y RNA fragments contribute to macrophage responses in GI cancer, but do not establish a YBX1/m5C pathway. The first requirements are to identify the relevant fragment and carrier, demonstrate delivery at a biologically relevant dose, and distinguish donor-derived RNA from endogenous recipient RNA. Only then can binding, an early molecular effect, and a functional macrophage response be linked through selective perturbation and rescue. Endosomal RNA sensing and effects of other EV-associated or soluble components remain viable explanations.

Therapeutic proposals remain premature until the dominant mechanism and its contribution to antitumor immunity are established. A pathway that changes macrophage markers without changing immune function would not provide the same rationale for intervention. The value of the proposed experiments is to determine whether YBX1-dependent RNA recognition is involved, or whether a different mechanism better explains the observed response.
